# Lack of Increase in Muscle Mitochondrial Protein Synthesis During the Course of Aerobic Exercise and Its Recovery in the Fasting State Irrespective of Obesity

**DOI:** 10.3389/fphys.2021.702742

**Published:** 2021-08-02

**Authors:** Nathan Serrano, Lee Tran, Nyssa Hoffman, Lori Roust, Elena A. De Filippis, Chad C. Carroll, Shivam H. Patel, Katon A. Kras, Matthew Buras, Christos S. Katsanos

**Affiliations:** ^1^Center for Metabolic and Vascular Biology, Arizona State University, Scottsdale, AZ, United States; ^2^Alix School of Medicine, Scottsdale, AZ, United States; ^3^Department of Health and Kinesiology, Purdue University, West Lafayette, IN, United States; ^4^Department of Biostatistics, Mayo Clinic in Arizona, Scottsdale, AZ, United States

**Keywords:** exercise, mitochondria, protein synthesis, PGC-1α, myosin heavy chain

## Abstract

Acute aerobic exercise induces skeletal muscle mitochondrial gene expression, which in turn can increase muscle mitochondrial protein synthesis. In this regard, the peroxisome proliferator-activated receptor γ coactivator 1α (PGC-1α), is a master regulator of mitochondrial biogenesis, and thus mitochondrial protein synthesis. However, PGC-1α expression is impaired in muscle of humans with obesity in response to acute aerobic exercise. Therefore, we sought to determine whether muscle mitochondrial protein synthesis is also impaired under the same conditions in humans with obesity. To this end, we measured mitochondrial and mixed-muscle protein synthesis in skeletal muscle of untrained subjects with (body fat: 34.7 ± 2.3%) and without (body fat: 25.3 ± 3.3%) obesity in a basal period and during a continuous period that included a 45 min cycling exercise (performed at an intensity corresponding to 65% of heart rate reserve) and a 3-h post-exercise recovery. Exercise increased PGC-1α mRNA expression in muscle of subjects without obesity, but not in subjects with obesity. However, muscle mitochondrial protein synthesis did not increase in either subject group. Similarly, mixed-muscle protein synthesis did not increase in either group. Concentrations of plasma amino acids decreased post-exercise in the subjects without obesity, but not in the subjects with obesity. We conclude that neither mitochondrial nor mixed-muscle protein synthesis increase in muscle of humans during the course of a session of aerobic exercise and its recovery period in the fasting state irrespective of obesity.

**Trial Registration:** The study has been registered within ClinicalTrials.gov (NCT01824173).

## Introduction

Mitochondria have primary role in supporting the energy needs of skeletal muscle, as well as other biological processes, such as enhancing recovery of muscle mass following muscle disuse (Zhang et al., 2018). In humans with obesity, dysfunction of muscle mitochondria has been implicated in muscle pathophysiology, including insulin resistance (Montgomery and Turner, 2015). Protein synthesis is important for maintaining proteostasis in tissues ensuring renewal of any given protein pool (Balch et al., 2008). In terms of mitochondrial proteostasis, sufficient mitochondrial protein synthesis ensures healthy skeletal muscle mitochondrial proteome, and because of that organ and whole-body metabolic health (Moehle et al., 2019). Current evidence indicates that mitochondrial protein synthesis is reduced in skeletal muscle of humans with obesity (Guillet et al., 2009; Tran et al., 2018). In this regard, approaches that upregulate mitochondrial protein synthesis in muscle are expected to support proteostasis and maintain mitochondrial proteome quantity and quality in muscle.

Mitochondrial protein synthesis in muscle increases in response to acute exercise in the fed state (Wilkinson et al., 2008; Donges et al., 2012). It is possible, however, that under these conditions increase in mitochondrial protein synthesis by aerobic exercise is simply the result of the provision of nutrients associated with the fed state and not the result of the exercise stimulus alone. This is because, increase in plasma amino acids alone increases muscle mitochondrial protein synthesis (Tran et al., 2018). On the other hand, there is evidence showing that acute aerobic exercise alone, and in the absence of provision of nutrients, provides adequate stimulus to increase protein synthesis in muscle of the mixed-muscle (i.e., overall) protein pool (Carraro et al., 1990; Harber et al., 2010; Mascher et al., 2011). Thus, aerobic exercise alone may increase also the mitochondrial protein synthesis in muscle.

In addition to the evidence showing upregulation of mitochondrial protein synthesis in skeletal muscle in the fed state (Wilkinson et al., 2008; Donges et al., 2012), prolonged aerobic exercise increases muscle mitochondrial protein synthesis in the fasting state (Konopka et al., 2017). However, there is evidence indicating lack of immediate increase in mitochondrial protein synthesis, despite increase in myofibrillar protein synthesis, in muscle by acute aerobic exercise in the fasting state (Di Donato et al., 2014). In the latter report, the impaired response may have been the result of insufficient exercise stimulus to upregulate peroxisome proliferator-activated receptor γ coactivator 1α (PGC-1α) in skeletal muscle. Rapid increase in gene expression in skeletal muscle in response to acute exercise enhances translational processing and elevates protein synthesis in muscle (Egan and Zierath, 2013). PGC-1α has central role in regulating mitochondrial biogenesis (Ventura-Clapier et al., 2008; Kang and Li Ji, 2012; Halling and Pilegaard, 2020), and thus upregulation of PGC-1α by exercise may be necessary to acutely increase mitochondrial protein synthesis in muscle as a result of aerobic exercise. In this regard, increase in mitochondrial protein synthesis in muscle is observed concomitant with increase in PGC-1α gene expression in muscle (Donges et al., 2012).

Although expression of PGC-1α increases immediately in response to aerobic exercise (Wright et al., 2007; De Filippis et al., 2008; Harber et al., 2010; Donges et al., 2012), the same response is not evident in muscle of humans with obesity (De Filippis et al., 2008). Based on this evidence, and the central role of PGC-1α gene expression in regulating mitochondrial biogenesis (Ventura-Clapier et al., 2008; Kang and Li Ji, 2012; Halling and Pilegaard, 2020), we hypothesized that muscle mitochondrial protein synthesis increases during the collective time period that includes aerobic exercise and post-exercise recovery in humans without obesity, but not in humans with obesity.

## Materials and Methods

### Subjects

Prior to obtaining written consent, the purpose of the study, requirements, and associated risks stemming from study participation were discussed with each study participant. The Institutional Review Board at Mayo Clinic approved all study procedures. We studied thirteen physically inactive subjects. The subjects did not participate in regular physical activity/exercise more than 2 days per week, and did not meet current physical activity recommendations (Piercy et al., 2018). Current consensus classifies physically inactive individuals as those that do not meet current physical activity recommendations (Tremblay et al., 2017). Determination of the physical activity levels was based on series of questions that included the type of physical activity, the number of times/days per week performed, the duration per session, the level of effort/intensity, as well as the number of months/years the subject has been performing the physical activity.

We sought to study subjects that differed in terms of obesity/adiposity. For that reason, we used the body mass index (BMI) as a screening tool to identify and enroll subjects with and without obesity. Subjects included in the study were 3 females and 3 males with BMI ranging between 32 and 40 kg/m^2^ (i.e., subjects with obesity; 34.7 ± 2.3% body fat), and 3 females and 4 males with BMI ranging between 19 and 27 kg/m^2^ (i.e., subjects without obesity; 25.3 ± 3.3% body fat), who served as study controls.

Subjects were invited to the Clinical Studies Infusion Unit (CSIU) at Mayo Clinic in Scottsdale, Arizona for screening. During that visit, we estimated insulin sensitivity from the plasma glucose and insulin responses to a 2-h oral glucose tolerance test (OGTT), and by calculating the Matsuda Insulin Sensitivity Index (ISI) (Matsuda and DeFronzo, 1999). We excluded subjects with evidence of diabetes (i.e., fasting plasma glucose ≥ 126 mg/dl, 2-h plasma glucose ≥ 200 mg/dl). All subjects enrolled in the study were classified as healthy based on medical history, standard physical exam, and blood and urine tests. Subjects included in the study following the screening visit to the CSIU, returned on a separate day to have their body composition determined, followed by an exercise test to measure their maximal oxygen uptake (VO_2_max). Body composition was evaluated using bioelectrical impedance analysis (BIA 310e, Biodynamics Corp., Shoreline, WA), and waist-to-hip ratio was calculated from circumference measurements using standard procedures (WHO, 2011). To measure VO_2_max, subjects underwent an incremental cycle ergometer test to exhaustion. For this test, after a 5 min unloaded warm-up period, the work rate was increased by 20 watts/min, and while subjects asked to maintain a pedaling rate of 65 revolutions/min. During the VO_2_max we performed a 12-lead electrocardiogram and monitor the blood pressure and blood oxygen saturation of the subjects. Subjects were verbally encouraged to cycle until exhaustion (Wood et al., 2010), and all subjects reached a respiratory exchange ratio > 1.1 before the termination of the VO_2_max test (Howley et al., 1995). Expired gases during the VO_2_max test were monitored on a continuous basis using a metabolic cart (MedGraphics Metabolic Cart, Saint Paul, MN).

### Experimental Design

On a separate day, subjects performed a stable-isotope tracer infusion study in our CSIU to measure rates of protein synthesis in skeletal muscle before and after an acute bout of aerobic exercise. Subjects were instructed to arrive to the CSIU at ∼7 a.m. after an overnight fasting period and abstain from any exercise beyond normal daily physical activities for 3 days prior to the infusion study. Compliance with the fasting and physical activity instructions was verified prior to initiation of the infusion study. After that, we placed one catheter into an antecubital arm vein for a 9-h infusion of d_10_ leucine (L-[2,3,3,4,5,5,5,6,6,6-^2^H_10_]leucine), at a rate of 0.15 μmol⋅kg FFM^–1^⋅min^–1^ (priming dose, 6.4 μmol⋅kg FFM^–1^) to measure rates of protein synthesis using the experimental procedures we have previously described (Tran et al., 2015). A separate catheter was placed in a retrograde fashion in a dorsal hand vein for blood sampling using the heated-hand technique.

We collected biopsy samples (∼100 mg) from the vastus lateralis muscle at 120 and 300 min following the start of the d_10_ leucine infusion, and while subjects were resting in bed (i.e., Basal). Muscle for the isolation of muscle mitochondria was processed immediately, while the remaining muscle was blotted dry and cleaned of any visible fat and connective tissue before storing the sample in liquid nitrogen. A single session of aerobic exercise was initiated soon after the second muscle biopsy. For the exercise session subjects cycled for 45 min at an intensity corresponding to 65% of their heart rate reserve. The latter was calculated from the subject’s resting heart rate and the maximal heart rate achieved during the VO_2_max test (Garber et al., 2011). During the exercise session, the workload on the cycle ergometer was adjusted as needed so that the subject kept their heart rate during the exercise within ± 5 bpm of the exercise target heart rate. Following the end of the exercise session, subjects rested in bed and until the end of the infusion study. A muscle biopsy was collected at the end of the infusion study, corresponding to 3.25 h after the end of the exercise session (i.e., Exercise), and by following procedures similar to those prior to the exercise session. In a single non-obese subject, we were not able to collect sufficient amount of muscle for the isolation of mitochondria at the end of the infusion study, and therefore that subject was not included in the analyses of the mitochondrial protein synthesis data.

We collected blood samples for the determination of blood d_9_-leucine enrichment during the Basal, exercise, and post-exercise recovery periods. Blood samples were collected at the following time points after the start of the d_10_-leucine infusion (min), corresponding to the Basal period: 110, 115, 140, 260, 280, 300, the exercise period: 315, 330, and post-exercise recovery: 355, 495, 515, 530. We have previously validated the use of blood d_9_-leucine enrichment measured at the selected time points to quantity protein fractional synthesis rate in skeletal muscle (Tran et al., 2015). Blood sampling at these time points was also used to determine blood chemistry parameters of interest in the course of the infusion study.

### Stable Isotope Enrichment Determination

D_9_-leucine enrichment was measured in blood, mixed-muscle protein, as well as muscle mitochondrial protein, using procedures we have previously described (Tran et al., 2015, 2018). Briefly, proteins in 1 ml of blood were precipitated using sulfosalicylic acid (SSA). Proteins in mixed-muscle (∼10 mg) were also precipitated using SSA, and by homogenizing the tissue in SSA. Proteins in the blood and mixed-muscle samples were hydrolyzed with 6 N HCl. Amino acids from these samples were passed through cation-exchange columns to isolate amino acids, and d_9_-leucine enrichment was measured using liquid chromatography-tandem mass spectrometry (LC-MS/MS).

D_9_-leucine enrichment of mitochondrial protein was determined in samples containing mitochondria isolated from ∼70 mg of fresh muscle. Isolation of muscle mitochondria was performed using procedures we have previously described (Kras et al., 2016). Muscles blotted gently dry from blood, weighed, and minced with scissors in 9 volumes of ice-cold Solution I, containing: 100 mM KCl, 40 mM Tris-HCl, 10 mM Tris-Base, 5 mM MgCl2, 1 mM EDTA, 1 mM ATP (pH 7.5). They were then gently homogenized in this solution by hand using a ground glass-to-glass Potter-Elvehjem homogenizer (total of 5 passes). The homogenate was centrifuged at 800 g for 10 min to obtain a supernatant containing mitochondria. The sample was then centrifuged at 14,000 g for 10 min to obtain a mitochondrial pellet. After discarding the supernatant, the mitochondrial pellet was re-suspended in 0.5 ml of Solution II, containing :100 mM KCl, 40 mM Tris–HCl, 10 mM Tris–Base, 5 mM MgCl2, 1 mM EDTA, and 0.2 mM ATP (pH 7.5). Following centrifugation at 7,000 g for 10 min, the supernatant was discarded and the mitochondrial pellet was re-suspended again in 0.5 ml of Solution II. After a final centrifugation at 4,000 g for 10 min and removal of the supernatant, the final mitochondrial pellet was re-suspended in a mannitol-sucrose buffer containing 220 mM Mannitol, 70 mM Sucrose, 10 mM Tris–HCl, and 1 mM EGTA (pH 7.4). All procedures for the isolation of muscle mitochondria were carried out on ice (i.e., homogenization) or at 4°C (i.e., centrifugation). This sample was then stored (at −80°C) for later determination of mitochondrial protein stable isotope enrichment. For the determination of the muscle mitochondrial protein stable isotope enrichment, the sample was hydrolyzed with 6 N HCl and d_9_-leucine enrichment was measured by LC-MS/MS, following the same procedures as for mixed-muscle protein.

### mRNA Quantification

We determined mRNA expression of genes of interest in the first (i.e., Basal) and last (i.e., reflecting the effects of exercise) muscle biopsies during the infusion study. mRNA expression was determined after extraction of total RNA from ∼20 mg muscle, and following procedures we have previously described (Tran et al., 2016, 2018). Genes of interest included that of PGC-1α, which regulates mitochondrial protein synthesis, as well as those encoding isoforms of the myosin heavy chain (MHC) protein, which constitutes the largest single protein pool in skeletal muscle. In this regard, MHC protein reflects major bulk of mixed-muscle protein whose synthesis rate is an end-point in the present study. With respect to the MHC genes, these include that of MHC-I (i.e., MYH7), MHC-IIa (i.e., MYH2) and MHC-IIx (i.e., MYH1). RNA isolated from muscle was used to synthesize cDNA using the ABI High Capacity cDNA Reverse Transcription kit (Thermo Fisher Scientific Inc., Waltham, MA). We used predesigned TaqMan^®^ gene expression assays (probe/primer sets) for PGC-1α (assay Hs00173304_m1), MYH7 (Hs00165276_m1), MYH2 (Hs00430042_m1), MYH1 (Hs_00428600_m1), and ACTB (Hs01060665_g1) (Thermo Fisher Scientific Inc., Waltham, MA). ACTB was employed as reference gene for normalizing the expression of the genes of interest. Real-time PCR was performed on an Applied Biosystems 7900HT Fast Real-Time PCR System (Thermo Fisher Scientific Inc., Waltham, MA).

### Other Blood Chemistry Assays

Blood samples analyzed in the assays below were collected prior to the exercise (i.e., Basal), within 10 min of the end of exercise [Post-EX (0 h)], and at the end of the infusion study [Post-EX (3 h)]. Concentrations of individual amino acids in plasma were analyzed by high-performance liquid chromatography (HPLC) (Carroll et al., 2005). Plasma amino acids measured included glutamic acid, asparagine, serine, glutamine, glycine, arginine, alanine, tyrosine, threonine, tryptophan, methionine, valine, phenylalanine, isoleucine, leucine and lysine. Plasma amino acid concentrations were evaluated as concentrations of total plasma amino acids, as well as essential amino acids (EAA; threonine, tryptophan, methionine, valine, phenylalanine, isoleucine, leucine, lysine) and branched-chain amino acids (BCAA; valine, isoleucine, leucine), given that amino acids in the last two groups are primary stimuli for protein synthesis in muscle (Liu et al., 2001; Wolfe, 2002; Yoshizawa, 2004). Concentrations of glucose in plasma were measured using an automated glucose analyzer (YSI 2300, Yellow Springs, OH), while those of insulin were measured using a commercially available ELISA kit (80-INSHU-E01.1; ALPCO Diagnostics, Windham, NH). Concentrations of total non-esterified fatty acids in plasma, were evaluated at Basal, and by using an enzymatic, calorimetric assay [Wako NEFA-HR(2) kit; Wako Chemicals, Richmond, VA].

### Calculations

Calculation of the fractional synthesis rate (%⋅h^–1^) of protein in skeletal muscle was performed using the precursor-product approach (Tran et al., 2015). Responses of mRNA for the genes of interest were evaluated using the comparative CT method (i.e., ΔΔCt), and relative changes were expressed as fold change (i.e., 2^–ΔΔCT^).

### Statistical Analyses

Differences in subject characteristics and in basal state blood chemistry parameters were compared using unpaired *t*-test. A two-way (obesity status x time/exercise) repeated-measures analysis of variance (ANOVA) was used to test for main effects of obesity status and exercise. Pairwise comparisons were performed using the Sidak multiple comparisons test. Correlations between variables of interest were evaluated using the Pearson *r* correlation coefficient. All data are presented as mean ± standard error of the mean (SEM), and the significance level was set at *P* ≤ 0.05. Statistical analyses were performed using GraphPad Prism (version 8.4.3, GraphPad Software, La Jolla, CA).

## Results

### Subject Characteristics

As expected, and based on our study design, subjects in the group with higher BMI values had higher body fat percentage, and in a way that the two subject groups differed in terms of obesity (i.e., adiposity) status. Average values for anthropometric characteristics and blood chemistry parameters for the two subject groups are presented in Table 1.

**TABLE 1:** Subject characteristics.

	**Lean**	**Obese**
Age (years)	29.1 ± 3.3	30.5 ± 4.7
Weight (kg)	77.2 ± 5.7	95.1 ± 4.8*
BMI (kg⋅m^–2^)	25.6 ± 0.6	35.3 ± 1.2*
FFM (kg)	57.8 ± 6.1	62.3 ± 4.4
Body fat mass (%)	25.3 ± 3.3	34.7 ± 2.3*
VO_2_max (ml⋅min^–1^)	1,800 ± 223	2,289 ± 188
VO_2_max (ml⋅kgFFM^–1^⋅min^–1^)	39.5 ± 2.3	33.1 ± 2.0
Waist-to-hip ratio	0.83 ± 0.02	0.92 ± 0.03*
Fasting plasma glucose (mg⋅dl^–1^)	81.6 ± 2.8	83.8 ± 2.2
Fasting plasma insulin (uIU⋅ml^–1^)	4.5 ± 0.4	11.8 ± 4.5
Matsuda-ISI	8.0 ± 1.5	4.2 ± 0.8*
HOMA-IR	0.8 ± 0.2	2.4 ± 0.9
HbA1c (%)	5.3 ± 0.1	5.4 ± 0.1
Plasma triglycerides (mg⋅dl^–1^)	110.1 ± 26.9	103.3 ± 16.0
Plasma NEFA (mmol⋅l^–1^)	0.35 ± 0.07	0.42 ± 0.05
Total plasma cholesterol (mg⋅dl^–1^)	156.4 ± 6.5	180.5 ± 8.8*
Plasma HDL-cholesterol (mg⋅dl^–1^)	56.7 ± 6.4	53.0 ± 5.7
Plasma LDL-cholesterol (mg⋅dl^–1^)	77.7 ± 5.7	106.8 ± 10.3*
TSH (mIU⋅l^–1^)	1.5 ± 0.3	2.7 ± 0.4*

Values are mean ± SEM. BMI, body mass index; FFM, fat-free mass; VO_2_max, maximal oxygen uptake; Matsuda-ISI, Matsuda insulin-sensitivity index; HOMA-IR, homeostatic model assessment of insulin-resistance; NEFA, non-esterified fatty acids, HDL, high density lipoprotein; LDL, low density lipoprotein; TSH, thyroid-stimulating hormone. *P ≤ 0.05 versus Lean subjects.

### Plasma Amino Acids, Glucose, and Insulin

**FIGURE 1:**
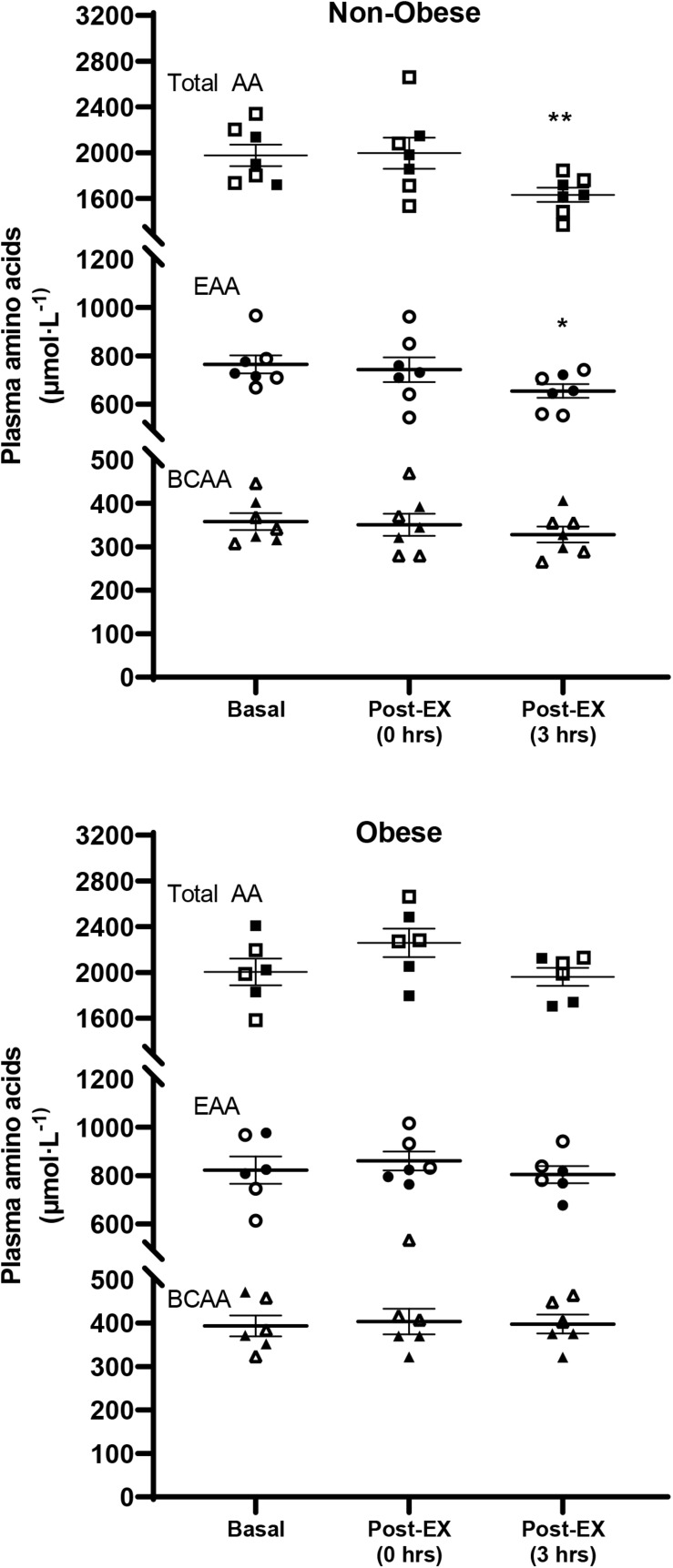
Plasma concentrations of total (Total AA), essential (EAA), and branched-chain (BCAA) amino acids, in the Basal study period (Basal), and immediately [Post-EX (0 h)] and at 3 h [Post-EX (3 h)] after the end of aerobic exercise in non-obese subjects (Non-obese; *n* = 7) and subjects with obesity (Obese; *n* = 6). Data presented are individual values from males (open symbols) and females (closed symbols), along with mean ± SEM. *P ≤ 0.05, **P ≤ 0.01 Basal versus Post-EX (3 h).

There was a significant exercise (*P* < 0.01), but not group, main effect for the plasma concentration of total amino acids. On the other hand, there was a significant group (*P* ≤ 0.05), but not exercise, effect for the plasma concentration of EAA, with EAA concentrations being higher across time in the subjects with obesity. No significant main effects for either group or exercise were detected for the plasma concentration of BCAA. Pairwise comparisons showed significant decrease in the concentration of total and EAA in the non-obese subject group, but not in the group of subjects with obesity (Figure 1).

Across groups, there was a significant main effect for plasma glucose concentration with exercise (*P* ≤ 0.05). Pairwise comparisons showed a trend for a significant decrease in plasma glucose concentration with exercise in the non-obese subject group (81.6 ± 2.8 versus 76.1 ± 4.0; *P* = 0.08), but not within the group of subjects with obesity (83.8 ± 2.2 versus 81.6 ± 3.3; *P* = 0.46). Across groups, there was a significant main effect for plasma insulin concentration with exercise (*P* ≤ 0.05). However, pairwise comparisons showed no significant difference between Basal and Exercise in either the non-obese subject group (4.5 ± 0.4 versus 3.8 ± 0.5; *P* = 0.10) or the group with subjects with obesity (11.8 ± 4.5 versus 10.7 ± 4.4; *P* = 0.22).

### Synthesis Rates of Mitochondrial and Mixed-Muscle Protein in Skeletal Muscle

**FIGURE 2:**
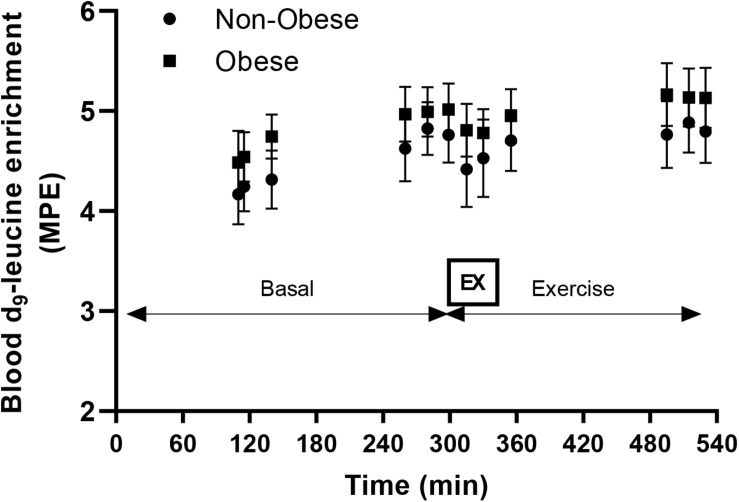
Enrichment of blood leucine with d_9_-leucine during the course of the infusion studies in the Basal (Basal) and Exercise (EX and post-EX recovery) study periods in non-obese subjects (Non-obese; *n* = 7) and subjects with obesity (Obese; *n* = 6). MPE, molar percent excess. EX: cycling exercise for 45 min at 65% of heart rate reserve.

**FIGURE 3:**
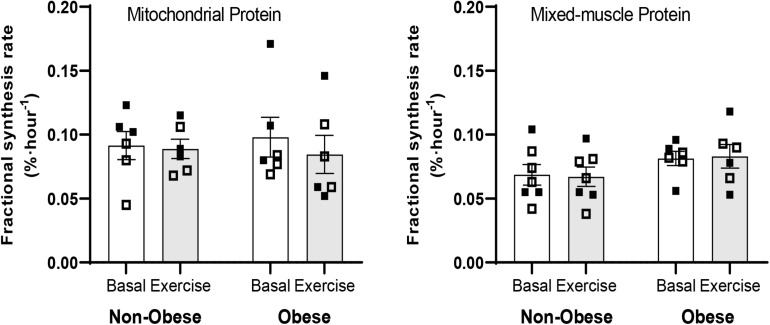
Fractional synthesis rates of mitochondrial and mixed-muscle proteins in skeletal muscle in the Basal (Basal) and Exercise (EX and post-EX recovery) study periods in non-obese subjects [Non-obese; *n* = 7 (*n* = 6 for mitochondrial protein)] and subjects with obesity (Obese; *n* = 6). Data presented are individual values from males (open symbols) and females (closed symbols), along with mean ± SEM.

Blood d_9_-leucine enrichments at Basal and during the Exercise study periods are shown in Figure 2. There were no main effects for group or exercise for either mitochondrial or mixed-muscle protein synthesis rates. Accordingly, pairwise comparisons indicated no differences within or between groups for either mitochondrial or mixed-muscle protein synthesis rates (Figure 3). Across study subjects, there was a trend for significant correlation between plasma EAA (*r* = 0.51; *P* = 0.07) and BCAA (*r* = 0.50; *P* = 0.08), but not total amino acids (*r* = 0.17; *P* = 0.57), measured at the end of the infusion study [Post-EX (3 h)] and the synthesis rates of mixed-muscle protein during the collective time period of the aerobic exercise and post-exercise recovery. Corresponding correlations of EAA and BCAA with synthesis rates of muscle mitochondrial protein were not significant (*P* = 0.60 and *P* = 0.88, respectively).

### Muscle mRNA Expression

**FIGURE 4:**
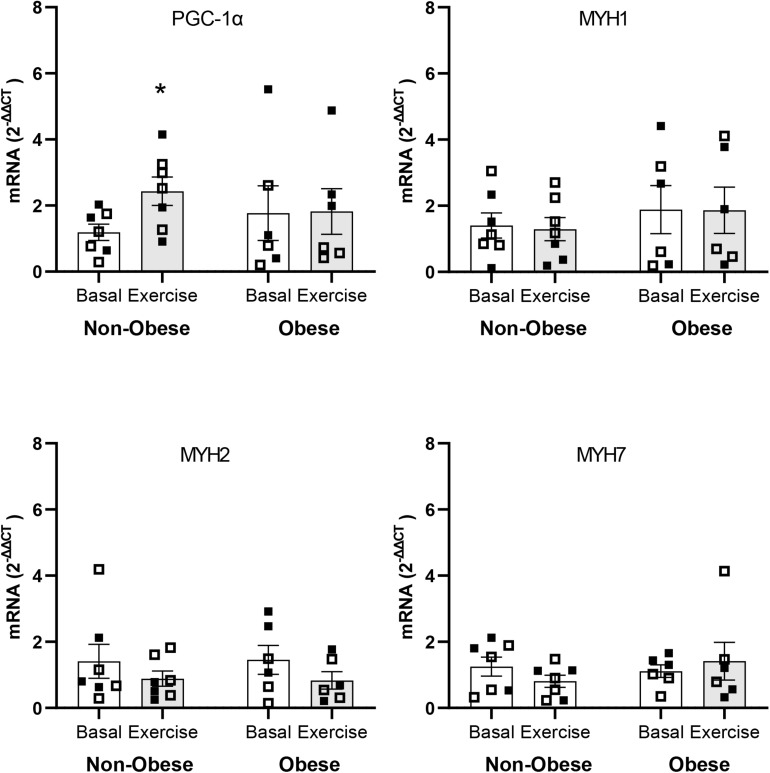
mRNA expressions of peroxisome proliferator-activated receptor γ coactivator 1α gene (i.e., PGC-1α) and myosin heavy chain genes (i.e., MYH1, MYH2, and MYH7) in skeletal muscle in the Basal study period (Basal) and at 3 h after the end of aerobic exercise (Exercise) in non-obese subjects (Non-obese; *n* = 7) and subjects with obesity (Obese; *n* = 6). Data presented are individual values from males (open symbols) and females (closed symbols), along with mean ± SEM. *P ≤ 0.05 for Basal versus Exercise.

Multiple comparison analyses indicated significant increase of more than twofold (2^–ΔΔCT^ = 2.17; *P* ≤ 0.05) in PGC-1α mRNA expression with exercise within the non-obese subject group. The corresponding increase in PGC-1α mRNA expression with exercise within the group of subject with obesity was not significant (2^–ΔΔCT^ = 1.25; *P* = 0.50). No differences were detected with respect to the mRNA responses of the genes regulating the expressions of the MHC isoforms in muscle either within or between groups. mRNA expression of all genes analyzed are shown in Figure 4.

## Discussion

Our study was designed to evaluate mitochondrial protein synthesis in skeletal muscle in the basal state and during the collective time period that includes aerobic exercise and post-exercise recovery, and in the absence of food intake. This is because provision of nutrients, including amino acids, alone is sufficient to stimulate mitochondrial protein synthesis in muscle regardless of obesity status (Beals et al., 2016, 2017; Tran et al., 2018). We show that neither muscle mitochondrial nor mixed-muscle protein synthesis are stimulated during the course of a session of aerobic exercise and its 3-h post-exercise recovery period in the fasted state.

Lack of increase in mitochondrial protein synthesis by exercise in the present study is in line with findings showing that mitochondrial protein synthesis does not increase immediately after exercise (Di Donato et al., 2014). Stimulation of gene expression by acute exercise in skeletal muscle is a mechanism to increase production of protein in muscle (Egan and Zierath, 2013). In this regard, PGC-1α gene expression has central role in increasing mitochondrial biogenesis (Ventura-Clapier et al., 2008; Kang and Li Ji, 2012; Halling and Pilegaard, 2020). In line with previous findings (De Filippis et al., 2008), we show that subjects with obesity have impaired PGC-1α mRNA expression in muscle in response to acute exercise. Impaired muscle PGC-1α gene expression in subjects with obesity may have played role in the observed lack of increase in muscle mitochondrial protein synthesis by exercise in these subjects. However, and despite increase in muscle PGC-1α gene expression, muscle mitochondrial protein synthesis did not increase in the subjects without obesity. Lack of increase in mitochondrial protein synthesis despite increase in PGC-1α gene expression by exercise in the present study points to disassociation between PGC-1α gene expression and mitochondrial protein synthesis in skeletal muscle. It is reasonable to conclude that increase in PGC-1α gene expression alone is not sufficient, or necessary as previously reported (Donges et al., 2012), to increase mitochondrial protein synthesis in muscle in response to acute aerobic exercise.

Alternatively, failure of mitochondrial protein synthesis to increase despite increase in PGC-1α gene expression by acute exercise in the muscle of non-obese subjects, may also be explained by the concomitant decrease in the concentration of plasma amino acids following exercise. Previous research also shows decrease in the concentrations of plasma amino acids during the post-exercise period (Struder et al., 1999; Makhro et al., 2016). The present study describes effects observed on muscle protein synthesis and was not designed to evaluate mechanisms contributing to the lack of increase in muscle protein synthesis by aerobic exercise. However, it is possible that the decrease in the concentration of plasma amino acids in response to exercise in the present study may have played role in preventing increase in protein synthesis by exercise. Relevant evidence indicates that regulation of protein synthesis in muscle depends on the sensing of the concentration of extracellular amino acids (Bohe et al., 2003), and that acute reduction in plasma amino acid concentrations below their basal levels inhibits muscle protein synthesis (Kobayashi et al., 2003). Therefore, decrease in the concentrations of plasma amino acids post-exercise in the present study may have prevented potential stimulation of muscle mitochondrial protein synthesis by the exercise-induced increase in muscle PGC-1α gene expression in the subjects without obesity.

Previous research suggests that mixed-muscle protein synthesis increases in response to acute aerobic exercise in a non-fed state (Carraro et al., 1990; Harber et al., 2010). This increase in mixed-muscle protein synthesis, however, is observed in a period that includes only the recovery period of exercise. Also, in the latter reports there is no evidence on whether increase in muscle protein synthesis in response to exercise occurred in the presence of an exercise-induced decrease in the concentration of plasma amino acids. In a separate report, protein synthesis in muscle did not increase post-exercise even in the presence of unchanged plasma amino acid concentrations, and as indicated by the plasma concentration of at least the amino acid phenylalanine (Sheffield-Moore et al., 2004). In this regard, findings from the latter study are in line with those in the present study with respect to mixed-muscle protein synthesis. Furthermore, the exercise did not upregulate the gene expression for proteins in the MHC protein pool, which forms the largest single protein pool in muscle, and whose increase in gene expression in response to exercise can ultimately mediate exercise-induced increase in the synthesis rate of mixed-muscle protein. Lack of response in MHC isoforms gene expression along with decrease in plasma amino acid concentrations, are indicative of absence of available biological stimuli in response to aerobic exercise that can upregulate protein synthesis in muscle in the fasting state.

Interestingly, plasma amino acid concentrations did not decrease in response to exercise in the subjects with obesity. To our knowledge, there are no other reports contrasting acute effects of aerobic exercise on plasma amino acid concentrations between humans with and without obesity. It is possible that a physiological increase in amino catabolism and use of amino acids as source of fuel in muscle during exercise (Wagenmakers, 1998; Overmyer et al., 2015) is less pronounced in the metabolic context of obesity, and where there is increased availability of fatty acids for oxidation (Holecek, 2020). Alternatively, downregulation of the expression of amino acid-catabolizing enzymes in liver and adipose tissue in obesity can mediate a pathological circumstance that increases the concentration of amino acids in plasma in humans with obesity (She et al., 2007; Wiklund et al., 2016), and which may counteract a physiological increase in amino acid catabolism in response to exercise in these individuals. Regardless the mechanism(s) implicated in this response, our findings provide original evidence showing altered plasma amino acid response to acute aerobic exercise in humans with obesity.

In the present study, protein synthesis in muscle reflects the collective response across both the exercise and post-exercise periods. It is possible that combining the exercise with the post-exercise recovery period may have diluted an acute increase in muscle protein synthesis occurring at some time during the post-exercise recovery. However, from a practical perspective it is the integrated response of muscle protein synthesis over time that is reflective of actual protein anabolism in muscle, and when compared to short-lived responses in muscle protein synthesis. In this regard, although there is an apparent increase in protein synthesis in muscle during the early period (i.e., first hour) after aerobic exercise, protein synthesis in muscle is not different from basal over 3 h post-exercise (Sheffield-Moore et al., 2004). Therefore, our study describes an integrated response over the course of aerobic exercise and post-exercise recovery.

We studied a relatively small number of subjects, and from both genders given that there is no evidence for gender differences in muscle protein synthesis (Markofski and Volpi, 2011). In this regard, our study is limited in its capacity to make conclusions in regards to the measured responses in males versus females. We chose an exercise intensity of 65% of heart rate reserve, which allowed subjects to successfully complete the continuous 45-min cycling exercise. However, we cannot exclude the possibility that higher exercise intensity may result in increased synthesis rates of mitochondrial and/or mixed muscle proteins. Also, it is possible that mitochondrial protein synthesis may increase at a time beyond the completion of the measurements of the present study. However, from a practical perspective, it is unlikely to abstain from food intake for several hours after exercise. It is already known that mixed-muscle protein synthesis increases post-exercise in the fed-state (Harber et al., 2010). When the latter evidence is consider along our findings that acute exercise decreases the concentration of plasma amino acids, it can be argued that dietary increase in plasma amino acids after aerobic exercise is necessary in order to observe increase in mitochondrial protein synthesis in response to exercise.

## Conclusion

We show that mitochondrial protein synthesis does not increase during the course of a session of aerobic exercise and its 3-h post-exercise recovery period in the fasting state. Impaired PGC-1α gene expression in muscle may play role in this response in humans with obesity. However, in humans without obesity, lack of increase in mitochondrial protein synthesis by acute aerobic exercise may result from an exercise-induced decrease in the concentration of plasma amino acids.

## Data Availability Statement

The original contributions presented in the study are included in the article, further inquiries can be directed to the corresponding author.

## Ethics Statement

The studies involving human participants were reviewed and approved by the Mayo Clinic Institutional Review Board. The subjects provided their written informed consent to participate in this study.

## Author Contributions

CSK and LR contributed to conception and design of the study. LT, NH, LR, ED, and CSK conducted the experiments and contributed to collection of muscle and blood samples. NS, LT, NH, CC, SP, and KK processed samples and analyzed the data. MB and CSK contributed to statistical analyses. NS wrote the first draft of the manuscript. All authors contributed to manuscript read, revision, and approved the submitted version.

## Conflict of Interest

The authors declare that the research was conducted in the absence of any commercial or financial relationships that could be construed as a potential conflict of interest.

## Publisher’s Note

All claims expressed in this article are solely those of the authors and do not necessarily represent those of their affiliated organizations, or those of the publisher, the editors and the reviewers. Any product that may be evaluated in this article, or claim that may be made by its manufacturer, is not guaranteed or endorsed by the publisher.
